# Serum Systemic Autoantibodies in Anti-N-Methyl-D-Aspartate Receptor Encephalitis

**DOI:** 10.3389/fneur.2020.00117

**Published:** 2020-02-28

**Authors:** Bingjun Zhang, Yu Yang, Yinyao Lin, Lulu Ai, Xuejiao Men, Zhengqi Lu

**Affiliations:** Department of Neurology, The Third Affiliated Hospital of Sun Yat-sen University, Guangzhou, China

**Keywords:** anti-N-methyl-D-aspartate receptor encephalitis, systemic autoantibodies, outcome, modified rankin scale, autoimmune diseases

## Abstract

**Objective:** The aim of this retrospective study was to investigate the relationship between serum systemic autoantibodies and anti-N-methyl-D-aspartate receptor (NMDAR) encephalitis.

**Methods:** Thirty-nine patients with anti-NMDAR encephalitis were examined for serum systemic autoantibodies (antinuclear antibodies, extractable nuclear antigen autoantibodies, rheumatoid factors, and anti-neutrophil cytoplasmic antibodies), in comparison with 39 neuromyelitis optica spectrum disorder (NMOSD) and 78 healthy controls. Clinical features, cerebrospinal fluid characteristics, and outcomes were compared between the two subgroups of anti-NMDAR patients with positive and negative systemic autoantibodies, respectively.

**Results:** Anti-NMDAR encephalitis patients had higher frequency of positive serum systemic autoantibodies than healthy controls (23.1 vs. 2.6%, *p* = 0.001) and lower frequency than NMOSD (23.1 vs. 48.7%, *p* = 0.018). No patients were diagnosed comorbidities with non-organ-specific autoimmune diseases. Consciousness disturbance was more frequent in autoantibodies positive group than in the negative group (88.9 vs. 40.0%, *p* = 0.02). Autoantibody positive group had a poorer outcome than autoantibody negative group (55.6 vs. 86.7%, *p* = 0.043). There was a negative correlation between serum autoantibodies and outcomes in anti-NMDAR encephalitis patients (*r* = −0.325, *p* = 0.044).

**Conclusion:** Our data demonstrated serum systemic autoantibodies were more frequent in anti-NMDAR encephalitis patients than in healthy controls and less frequent than NMOSD, which were associated with higher severity of disease.

## Introduction

Anti-N-methyl-D-aspartate receptor (NMDAR) encephalitis is the most common autoimmune encephalitis (AE) related to antibody-mediated synaptic dysfunction, which is characterized by the subacute development of psychosis, epileptic seizures, memory deficit, autonomic instability, and a decrease in the level of consciousness (1). The disease can be triggered by NMDAR-expressing ovarian teratomas or occur secondarily to virus encephalitis, while the initiating events remain unclear in most cases (2–4).

Previous reports have demonstrated the intertwining relationships between systemic immune abnormalities and immune-mediated neurological disorders, such as neuromyelitis optica spectrum disorder (NMOSD), and multiple sclerosis (5, 6). Recently, several studies have revealed anti-thyroid antibodies abnormalities and elevated complement levels are frequent in patients with anti-NMDAR encephalitis, which are associated with the outcomes (7–9). Serum systemic autoantibodies that are indicative of systemic immune diseases, such as antinuclear antibodies (ANAs), extractable nuclear antigen autoantibodies (ENAs), rheumatoid factors (RFs), and anti-neutrophil cytoplasmic antibodies (ANCAs), have also been detected in patients with AE, but the literature in this regard is limited (8, 10). However, the association between systemic autoantibodies and anti-NMDAR encephalitis has not been discussed.

Therefore, this study aims to analyze serum systemic autoantibodies in patients with anti-NMDAR encephalitis, and determine the association with clinical characteristics in the patients.

## Methods

### Patients and Controls

This retrospective study enrolled 39 patients with anti-NMDAR encephalitis who were admitted to the Third Affiliated Hospital of Sun Yat-sen University from February 2016 to December 2017. Anti-NMDAR encephalitis was defined according to diagnostic criteria (11). A commercial indirect fluorescence assay (EUROIMMUN, Medizinische Labordiagnostika, Lubeck, Germany) was used to screen for IgG antibody to the NMDAR (9). The disease control group included 39 NMOSD patients who were defined according to diagnostic criteria (12). The healthy control group included 78 individuals who visited the health examination center at our hospital for health examination. The additional data of healthy control group was shown in Supplementary Material.

Gender, age, clinical manifestations, time from onset to hospitalization, length of hospital stay, and length of follow-up were recorded. Examinations including brain magnetic resonance imaging (MRI) and cerebrospinal fluid (CSF) were reviewed. The patients received first-line immunotherapy [steroids and intravenous immunoglobulin (IVIg) with or without plasma exchange], second-line immunotherapy (rituximab with or without cyclophosphamide). The patients' neurological function and outcome were evaluated using the modified Rankin Scale (mRS) at admission and at discharge.

### Autoantibodies Analysis in Serum

Systemic autoantibodies (ANAs, ENAs, RFs, ANCAs) tests were measured on Tenfly Phoenix Auto Blot Analyzer (YHLO, Shenzhen, China) at the clinical rheumatology immunology laboratory of the Third Affiliated Hospital of Sun Yat-sen University (6). The samples were collected at admission (prior to immune treatment), at discharge, and the last follow-up.

### Statistical Analysis

All quantitative data in this study are presented as mean ± standard deviation (SD) or median (range). Quantitative data were processed using the Mann-Whitney U-test or Student's *t*-test. Qualitative data were analyzed with the χ^2^ test or Fisher's exact test. The relationship of two variables was analyzed using Spearman's rank test. Values of *p* < 0.05 were considered statistically significant. Statistical analysis was performed by SPSS version 22.0 (SPSS Inc., Chicago, IL, USA).

## Results

### Demographic and Clinical Features

Baseline characteristics were presented in Table 1. A total of 39 patients with anti-NMDAR encephalitis (age, 27.7 ± 12.7 years; female: male, 19:20), 39 patients with NMOSD (age, 41.1 ± 13.8 years; female: male, 36:3), and 78 controls (age, 30.3 ± 12.2 years; female: male, 41:37) were enrolled in our study. Ten patients (25.6%) had ovarian teratoma. Other tumors were not detected. As for the clinical manifestation at onset, 21 patients (53.8%) had epileptic seizures, 29 patients (74.4%) had behavioral and psychiatric disturbances, 20 patients (51.3%) had consciousness disturbance, 9 patients (23.1%) had short-term memory deficits. Fourteen patients (35.9%) had abnormal MRI findings, and 25 patients (64.1%) had abnormal CSF findings. The median anti-NMDAR antibodies titer in CSF is 1:64 (1:1–1:320). On admission, the median mRS score which used to evaluate the severity of anti-NMDAR encephalitis was 4 (range 1–5). 24 patients (61.5%) received first-line treatment and 15 patients (38.5%) received first-line and second-line immunotherapy. 31 patients (79.5%) had a good outcome (mRS < 2) at discharge. Serum systemic autoantibodies were more frequent in anti-NMDAR encephalitis patients than in healthy controls (23.1 vs. 2.6%, *p* = 0.001), which less frequent in anti-NMDAR encephalitis patients than in NMOSD (23.1 vs. 48.7%, *p* = 0.018).

**Table 1:** Demographic features of anti-NMDAR encephalitis patients and controls.

**Characteristics**	**Anti-NMDAR encephalitis (*n* = 39)**	**NMOSD (*n* = 39)**	**Healthy control (*n* = 78)**	**p1**	**p2**	**p3**
F/M	19:20	36:3	41:37	<0.001	0.695	<0.001
Age (mean ± SD, years)	27.7 ± 12.7	41.1 ± 13.8	30.3 ± 12.2	<0.001	0.286	<0.001
Patients with ovarian teratoma, *n* (%)	10 (25.6)					
Clinical presentation at onset, *n* (%)						
Epileptic seizures	21 (53.8)					
Behavioral and psychiatric disturbances	29 (74.4)					
Consciousness disturbance	20 (51.3)					
Short-term memory deficits	9 (23.1)					
Brain lesions on MRI, *n* (%)	14 (35.9)					
CSF abnormalities, *n* (%)	25 (64.1)					
CSF NMDAR antibody, median (range)	1:64 (1:1–1:320)					
Serum systemic autoantibodies, *n* (%)	9 (23.1)	19 (48.7)	2 (2.6)	0.018	0.001	<0.001
mRS on admission, median (range)	4 (1–5)					
mRS < 2 at discharge, *n* (%)	31 (79.5)					
First-line treatment, *n* (%)	24 (61.5)					
First-line combined with second-line treatment, *n* (%)	15 (38.5)					

*CSF, cerebrospinal fluid; F, female; M, male; MRI, magnetic resonance imaging; mRS, modified Rankin Scale; NMDAR, N-methyl-D-aspartate receptor; NMOSD, Neuromyelitis optica spectrum disorder; SD, standard deviation*.

*p1, anti-NMDAR encephalitis vs. NMOSD; p2, anti-NMDAR encephalitis vs. healthy control; p3, NMOSD vs. healthy control*.

### Clinical Characteristics in Serum Autoantibodies Positive Patients With Anti-NMDAR Encephalitis

As shown in Table 2, 9 patients (age, 30.0 ± 16.2 years; female: male, 6:3) had positive serum autoantibodies, including 7 ANA positive, 1 ANCA positive, and 1 centromere antibody positive. However, no patients were diagnosed comorbidities with non-organ-specific autoimmune diseases [e.g., lupus, sjögren syndrome (SS), rheumatoid arthritis (RA)]. After treatment, 7 patients (77.8%) serum autoantibodies turn to negative at discharge. The serum autoantibodies results of all anti-NMDAR encephalitis patients had no alteration from discharge to the last follow-up.

**Table 2:** Clinical characteristics in anti-NMDAR encephalitis patients with serum autoantibodies positive.

**Patients**	**Gender**	**Age, years**	**Systemic autoantibodies**	**Turn negative**	**Good outcomes**	**CSF NMDAR antibody**
1	F	22	ANA (1:100)	Yes	Yes	1:100
2	M	10	ANA (1: 100)	No	No	1:1
3	F	50	ANA (1:100)	Yes	Yes	1:32
4	F	33	ANA (1:100)	No	No	1:320
5	F	31	ANA (1:100)	Yes	Yes	1:32
6	M	32	ANA (1:100)	Yes	No	1:64
7	F	13	ANA (1:100)	Yes	Yes	1:10
8	M	20	ANCA (1:100)	Yes	Yes	1:32
9	F	59	Centromere antibody (1:100)	Yes	No	1:320

*ANA, antinuclear antibody; ANCA, anti-neutrophil cytoplasmic antibody; CSF, cerebrospinal fluid; F, female; M, male; NMDAR, N-methyl-D-aspartate receptor*.

### Clinical Characteristics Between Serum Autoantibodies Positive and Negative Patients With Anti-NMDAR Encephalitis

**Figure 1:**
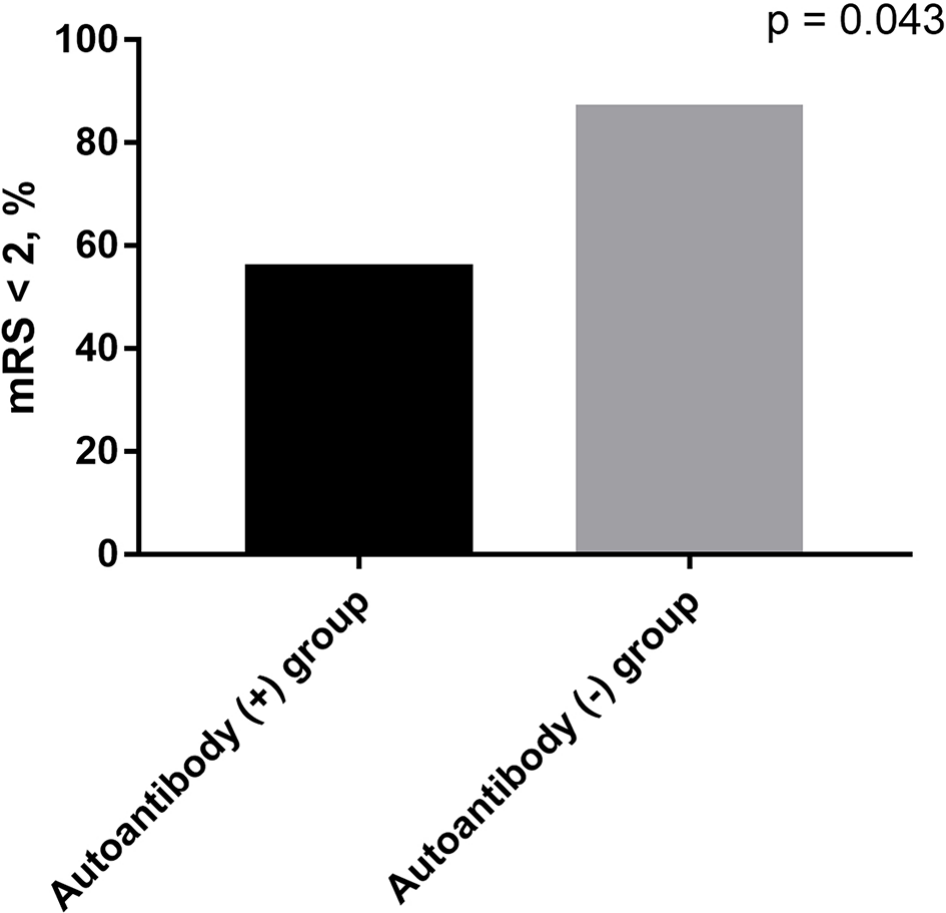
The outcomes in anti-NMDAR encephalitis patients. Autoantibody positive group had a poorer outcome than autoantibody negative group (55.6 vs. 86.7%, *p* = 0.043).

As shown in Table 3, there were no statistical differences in gender and age between serum autoantibodies positive and negative patients with anti-NMDAR encephalitis (*p* > 0.05). However, consciousness disturbance was more frequent in autoantibodies positive group than in the negative group (88.9 vs. 40.0%, *p* = 0.02). No difference was found in MRI findings (Table 4), CSF abnormalities, CSF anti-NMDAR antibodies titers, mRS on admission, time from onset to hospitalization, length of hospital stay, length of follow-up, and immune treatment between two groups (*p* > 0.05). Autoantibody positive group had a poorer outcome than autoantibody negative group (55.6 vs. 86.7%, *p* = 0.043) (Figure 1). The relationship between serum autoantibodies and outcome in patients with anti-NMDAR encephalitis was evaluated. There was a negative correlation between serum autoantibodies and outcome in anti-NMDAR encephalitis patients (*r* = −0.325, *p* = 0.044).

**Table 3:** Comparison of clinical features in patients with anti-NMDAR encephalitis based on serum systemic autoantibodies status.

**Characteristics**	**Serum autoantibodies positive (*n* = 9)**	**Serum autoantibodies negative (*n* = 30)**	***p***
F/M	6: 3	13: 17	0.273
Age (mean ± SD, years)	30.0 ± 16.2	27.0 ± 11.7	0.538
Patients with ovarian teratoma, *n* (%)	4 (44.4)	6 (20.0)	0.299
Clinical presentation at onset, *n* (%)			
Epileptic seizures	6 (66.7)	15 (50)	0.464
Behavioral and psychiatric disturbances	7 (77.8)	22 (73.3)	1
Consciousness disturbance	8 (88.9)	12 (40)	0.02
Short-term memory deficits	1 (11.1)	8 (26.7)	0.654
Brain lesions on MRI, *n* (%)	3 (33.3)	11 (36.7)	0.855
CSF abnormalities, *n* (%)	7 (77.8)	18 (60.0)	0.445
CSF NMDAR antibody, median (range)	1:32 (1:1–1:320)	1:64 (1:10–1:320)	0.476
mRS on admission, median (range)	4 (1–5)	4 (1–5)	0.48
Time from onset to hospitalization, days	20 (1–60)	15 (4–120)	0.611
Length of hospital stay, days	26 (15–84)	26 (10–108)	0.987
mRS < 2 at discharge, *n* (%)	5 (55.6)	26 (86.7)	0.043
First-line treatment, *n* (%)	5 (55.6)	19 (63.3)	0.711
First-line combined with second-line treatment, *n* (%)	4 (44.4)	11 (36.7)	0.711
Follow-up, months	8 (3–48)	10 (3–48)	0.588
Follow-up CSF NMDAR antibody, median (range)	1:10 (0–1:10)	1:1 (0–1:100)	0.883

*CSF, cerebrospinal fluid; F, female; M, male; MRI, magnetic resonance imaging; mRS, modified Rankin Scale; NMDAR, N-methyl-D-aspartate receptor; SD, standard deviation*.

**Table 4:** Comparison of MRI features in patients with anti-NMDAR encephalitis based on serum systemic autoantibodies status.

**Characteristics, *n* (%)**	**Serum autoantibodies positive (*n* = 9)**	**Serum autoantibodies negative (*n* = 30)**	***p***
Limbic system	2 (22.2)	4 (13.3)	0.903
Brain lobes	3 (33.3)	5 (16.7)	0.538
White Matter	1 (11.1)	1 (3.3)	0.947
Basal ganglia	1 (11.1)	0	0.517
Brainstem	1 (11.1)	1 (3.3)	0.947
Cerebellum	1 (11.1)	1 (3.3)	0.947

*MRI, magnetic resonance imaging; NMDAR, N-methyl-D-aspartate receptor*.

## Discussion

In the present study, we found that anti-NMDAR encephalitis patients had higher frequency of positive serum systemic autoantibodies than healthy controls and lower frequency than NMOSD. We also found ANA was the most common serum autoantibodies in anti-NMDAR encephalitis. However, no patients were diagnosed comorbidities with non-organ-specific autoimmune diseases. Furthermore, good outcomes in anti-NMDAR encephalitis patients were significantly negatively associated with the serum autoantibodies. To the best of our knowledge, this is the first study to analyze serum systemic autoantibodies in patients with anti-NMDAR encephalitis.

Autoantibodies are the result of a failure of the immune system to discriminate between “self” and “non-self,” which target a person's own antigen (13). Autoantibodies cause inflammation, damage, and/or dysfunction of organs, resulting in autoimmune disorders. Disorders due to systemic autoantibodies, which include ANAs, ENAs, RFs, and ANCAs, affect multiple organs or systems. Several previous reports have found the idiopathic inflammatory demyelinating diseases have positive systemic autoantibodies and comorbidities with systemic autoimmune disorders (5, 14). In our previous study, we found 25.8% NMOSD comorbidities with systemic or organ-specific autoimmune disorders (6). Half of NMOSD have ANA or Anti-SS-related antigen A antibody positive in the serum. Recently, anti-thyroid antibodies abnormalities and elevated complement levels are frequent in patients with anti-NMDAR encephalitis, which are associated with the short-term prognosis (7–9). Furthermore, in our present study, only 9 in 39 of anti-NMDAR encephalitis were positive for serum systemic autoantibodies (7 ANA, 1 ANCA, and 1 centromere antibody). However, the titers of serum systemic autoantibodies were low in the patients. There were no patients diagnosed comorbidities with systemic autoimmune diseases. Our results have some different from the recent paper conducted by Zhao et al., which found anti-NMDAR encephalitis can coexist with non-organ-specific autoimmune diseases, such as lupus (15). The paper also found that autoimmune diseases were more frequent in anti-leucine-rich glioma-inactivated 1 encephalitis than in anti-NMDAR encephalitis, and that autoimmune comorbidities did not affect the clinical course of AE. The prevalence of the most common type of autoantibody in the anti-NMDAR encephalitis, ANA, may be positive with a variety of autoimmune diseases, including lupus, SS, RA, and autoimmune hepatitis, and many non-autoimmune factors, such as tumors, infectious diseases, and pharmaceuticals (16, 17). Also, an ANA positive frequency of 2–20% in healthy individuals has been reported (18–20). In the present study, healthy control showed a relatively low frequency of 2.6% for positive systemic autoantibodies. Furthermore, anti-NMDAR encephalitis patients had higher frequency of serum autoantibodies than healthy controls. Autoimmune disease is caused by a complex genetic predisposition that is attributable to multiple genetic variants and human leukocyte antigen (HLA) alleles (21, 22). The phenomenons are not rare that one autoimmune disease increases the chance of an additional autoimmune disease, and that autoimmune disease patient has multiple positive antibodies. Recent studies have shown that anti-NMDAR encephalitis was associated with the HLA alleles (23, 24). Perhaps, genetic predisposition is the important reason for the increased prevalence of systemic autoantibodies in patients with anti-NMDAR encephalitis.

This study also compared the characteristics of anti-NMDAR encephalitis patients according to serum systemic autoantibodies status. In our study, more than one half of anti-NMDAR encephalitis patients had consciousness disturbance. And a study conducted by Lin et al. showed that consciousness disturbance is typically severe syndrome (7), which is consistent with our result. However, we found consciousness disturbance was more frequent in autoantibodies positive group than in negative group. Interestingly, there was a negative correlation between serum autoantibodies and outcome in anti-NMDAR encephalitis patients. It is easy for us to find a direct phenomenon in autoantibodies positive group: severe syndrome, bad outcome. The reasons for this are still unknown. One of the reasons is due to the profound derangement of the immune system in these severe patients with systemic autoantibodies. Positive systemic autoantibodies were detected in the CSF in neuropsychiatric lupus patients, which played important role in the disruption of blood brain barrier (BBB) (25–27). Recent studies have suggested the integrity of BBB was impaired in anti-NMDAR antibody positive patients (28, 29). Although systemic autoantibodies were not tested in the CSF in our study, it is thus tempting to speculate that serum systemic autoantibodies play a role in the damages of neurons by interacting with anti-NMDAR antibody against neuronal surface antigens, where they can access the brain because of BBB disruption. The synergistic effect between systemic autoantibodies and anti-NMDAR antibody might cause more severe symptoms, such as consciousness disturbance which resulted in higher mRS score.

Anti-NMDAR encephalitis is a treatable and even curable disease. In our present study, nearly 80% had good outcome (mRS < 2) at discharge, which is consistent with previous researches (1, 30). The main treatments for anti-NMDAR encephalitis are stepwise escalation of immunotherapy and tumor resection (1, 11). In this study, 24 patients (61.5%) received first-line treatment and 15 patients (38.5%) received first-line and second-line immunotherapies. Although the difference was not statistically significant, it seemed like more anti-NMDAR encephalitis patients with systemic autoantibodies receiving second-line treatments than that without systemic autoantibodies. That finding was consistent with clinical features that consciousness disturbance was more prevalent in patients with systemic autoantibodies, though the difference of mRS at admission was not statistically significant.

We are aware that this study has some limitations. First, the sample size was relatively small. Second, we only detected systemic autoantibodies in the serum, not in the CSF, which result could strengthen our findings. Third, as a retrospective study, bias is inevitable.

In conclusion, our data demonstrated serum systemic autoantibodies were more frequent in anti-NMDAR encephalitis patients than in healthy controls and less frequent than NMOSD, which were associated with higher severity of disease.

## Data Availability Statement

The raw data supporting the conclusions of this article will be made available by the authors, without undue reservation, to any qualified researcher.

## Ethics Statement

This study was approved by the Medical Ethics Committee of the Third Affiliated Hospital of Sun Yat-sen University. The need for obtaining written informed consent from patients or their family members was waived by the same committee.

## Author Contributions

BZ, YY, YL, and ZL designed the research. LA performed the experiments and analyzed the data. XM wrote the main manuscript text and prepared the figures. BZ edited and revised the manuscript. All authors reviewed and approved the manuscript.

### Conflict of Interest

The authors declare that the research was conducted in the absence of any commercial or financial relationships that could be construed as a potential conflict of interest.

## Supplementary Material

Supplementary Table 1 Additional data of healthy controls.
